# Fetal anomalies in gestational diabetes mellitus and risk of fetal anomalies in relation to pre-conceptional blood sugar and glycosylated hemoglobin

**DOI:** 10.34763/jmotherandchild.20222601.d-22-00040

**Published:** 2023-02-22

**Authors:** Rami M. M. Al-Shwyiat, Ahmed M. Radwan

**Affiliations:** Department of Obstetrics and Gynecology, King Hussain Royal Medical Services (KH-RMS), Jordan Egypt; Department of Obstetrics and Gynecology, Faculty of Medicine, Zagazig University, Sharkia, Egypt

**Keywords:** Fetal anomalies, gestational diabetes mellitus, pre-conceptional blood sugar, glycosylated hemoglobin

## Abstract

**Background:**

The risk of fetal anomalies (FAs) is increased in infants of diabetic mothers. FAs are closely related to the glycosylated hemoglobin (HbA1c) level in pregnancy.

**Objectives:**

To detect the prevalence of FAs in women with gestational diabetes mellitus (GDM).

**Material and methods:**

157 pregnant women with GDM were included in this study, and data from 151 women were analyzed. Beyond the regular antenatal check-up, the HbA1c was checked monthly during the antenatal follow-up. Collected data after delivery were analyzed to detect the prevalence of FAs in women with GDM and the risk of FAs in relation to the pre-conceptional blood sugar and HbA1c.

**Results:**

The FAs were recorded in 8.6% (13) of the 151 women with GDM. The recorded FAs were cardiovascular [2.6% (4)], musculoskeletal [1.3% (2)], urogenital [1.3% (2)], gastrointestinal [1.3% (2)], facial [0.7% (1)], central nervous system [0.7% (1)], and multiple FAs [0.7% (1)]. The uncontrolled pre-conceptional blood sugar significantly increased RR [RR 2.2 (95%CI: 1.7-2.9); P < 0.001], and odds of FAs [OR 17.05 (95%CI: 2.2-134.9); P = 0.007] in women with GDM. In addition, the HbA1c ≥6.5 significantly increased RR [RR 2.8 (95% CI: 2.1-3.8); P < 0.001], and odds of FAs [OR 24.8 (95% CI: 3.1-196.7); P = 0.002] in women with GDM.

**Conclusion:**

In this study, the prevalence of FAs in women with GDM was 8.6%. Uncontrolled pre-conceptional blood sugar and HbA1c ≥6.5 in the first trimester significantly increased the relative risk and the odds of FAs.

## Introduction

The incidence of gestational diabetes mellitus (GDM) is 2-9% [1, 2]. Diabetes during pregnancy is associated with increased risk of fetal anomalies (FAs) [3, 4].

An association between DM and FAs has been recorded since the 19^th^ century, and caudal regression syndrome has a strong association with DM. Studies of infants of diabetic mothers showed increased risk of cardiovascular system (CVS), genitourinary, and musculoskeletal FAs in infants of women with uncontrolled diabetes during pregnancy [5, 6].

Diabetic embryopathy is a spectrum of FAs or disruptions caused by maternal DM [7]. The risk of FAs, particularly open neural tube defect (ONTD) anomalies [8], is markedly increased in infants of diabetic mothers.

FAs are closely related to the glycosylated hemoglobin (HbA1c) level in pregnancy [9, 10]. Therefore, this prospective observational study was designed to detect the prevalence of FAs in women with GDM (primary outcome), and the risk of FAs in relation to the pre-conceptional blood sugar and HbA1c (secondary outcome).

## Patients and Methods

This prospective observational study was conducted over two years (August 2020 until August 2021) after approval of the Obstetrics and Gynecology department’s ethical committee of United Doctors Hospital, Jeddah, KSA.

Women discovered to have GDM during the antenatal screening (between 24-28 weeks’ gestation), with regular antenatal follow-up at least twice a month, were included in this study after informed consent in accordance with the Declaration of Helsinki.

Women with irregular antenatal care or incomplete antenatal records, and those who refused to participate, were excluded from this study.

157 pregnant women with GDM regularly attending the antenatal clinic were included in this study, and the data of 151 women were eventually analyzed (with 6 women being excluded because of incomplete antenatal records and absence of delivery outcome).

The American Diabetes Association recommends screening of all pregnant women at 24-28 weeks’ gestation for GDM using the OGTT (oral glucose tolerance test) [11].

GDM is a group of glucose intolerances discovered for the first-time during pregnancy and diagnosed according to the American Diabetes Association criteria [11].

In women with GDM, the diabetic diet regimen started to control blood glucose level. If the diabetic diet regimen gave unsatisfactory blood glucose control (pre-prandial >5.3 mmol/l, and 2-hours post-prandial >6.7 mmol/l), subcutaneous insulin was prescribed to control the blood glucose level (mainly insulin Monotard and insulin Actrapid as bolus injections).

During the antenatal follow-ups, HbA1c levels were checked monthly (normal HbA1c <5.7%) beside the regular antenatal check, which included urine sugar, urine albumin, blood pressure, fetal assessment, and basic laboratory investigations according to the hospital’s protocol.

Collected data after delivery were analyzed to detect the prevalence of FAs in women GDM (primary outcome), and the risk of FAs in relation to the pre-conceptional blood sugar and HbA1c (secondary outcome).

### Sample Size

The required sample size was calculated using G Power software version 3.1.9.4 for sample size calculation, setting α-error probability at 0.05, power (1- β error probability) at 0.95%, and effective sample size (w) at 0.5. An effective sample including ≥100 was needed to produce a statistically acceptable figure.

### Statistical Analysis

Collected data were statistically analyzed using the Statistical Package for Social Sciences (SPSS): computer software version 20 (Chicago, IL, USA). Numerical variables were presented as mean and standard deviation (±SD), while categorical variables were presented as number (n) and percentage (%). The odds ratio (OR) and relative risk (RR) of FAs in relation to the pre-conceptional blood sugar and HbA1c were calculated. *P*-value <0.05 was considered significant.

## Results

157 pregnant women with GDM attending the antenatal clinic were included in this study, and data of 151 [96.2%] were finally analyzed. The mean age of the studied women, history of medical disorders, HbA1c, daily insulin dose, and the obstetrics outcome are presented in Table 1.

**Table 1:** The maternal age, medical disorders, HbA1c, daily insulin dose, and the obstetrics outcome of the studied women with GDM

Variables	Studied population Number 151
**Maternal characteristics**	
Age (years)	26.9 ± 3.28
History of hypertension	11.9% (18/151)
Family history of diabetes	63.6% (96/151)

HbA1c (g%)	5.57 ± 0.91
Daily insulin dose	25.7 ± 19.03

**Obstetrics Outcome**	
Past history of Miscarriage	73.5% (111/151)
Cesarean section (CS)	30.5% (46/151)
Gestational age at delivery (weeks)	37.8 ± 0.6
Birth weight (kg)	3.26 ± 0.2

Data presented as number (n), percentage (%) or mean and Standard deviation (±SD) HbA1c: Glycosylated hemoglobin

FAs were recorded in 8.6% (13/151) of the studied women with GDM. The recorded FAs were cardiovascular [transposition of great arteries (TGA), ventricular septal defect (VSD), and atrial septal defect (ASD)] in 2.6% (4); musculoskeletal [caudal dysgenesis, and limb reduction] in 1.3% (2); urogenital [renal agenesis, and hydronephrosis] in 1.3% (2); gastrointestinal [pyloric stenosis and duodenal atresia] in 1.3% (2); facial [cleft lip and palate] in 0.7% (1); central nervous system [(ONTD) spina bifida] in 0.7% (1); and multiple FAs in 0.7% (1) [Table 2].

**Table 2:** The fetal anomalies (FAs) recorded in studied women with GDM

Fetal anomalies (FAs)	Number and (%)
Cardiovascular system (TGA, VSD, ASD)	(4/151) 2.6%
Musculoskeletal system (caudal dysgenesis and limb reduction)	(2/151) 1.3%
Urogenital anomalies (renal agenesis and hydronephrosis)	(2/151) 1.3%
Gastrointestinal system (pyloric stenosis and duodenal atresia)	(2/151) 13%
Facial anomalies (cleft lip and palate)	(1/151) 0.7%
Central nervous system (ONTD (spina bifida))	(1/151) 0.7%
Multiple FA anomalies	(1/151) 0.7%
Total	(13/150) 8.6%

ASD: Atrial septal defect. Data presented as number (n) and percentage (%). GDM: Gestational diabetes mellitus. ONTD: Open neural tube defect. TGA: Transposition of the great arteries. VSD: Ventricular septal defect.

The uncontrolled pre-conceptional blood sugar significantly increased RR [RR 2.2 (95%CI: 1.7-2.9); *P* < 0.001], and odds of FAs [OR 17.05 (95%CI: 2.2-134.9); *P* = 0.007], in women with GDM.

In addition, the HbA1c ≥6.5 significantly increased RR [RR 2.8 (95% CI: 2.1-3.8); P < 0.001], and odds of FAs [OR 24.8 (95% CI: 3.1-196.7); *P* = 0.002], in women with GDM [Table 3].

**Table 3:** The RR and OR of FAs in relation to the pre-conceptional blood sugar and HbA1c

Studied women with GDM (N = 151)	FAs group Exposed group (N = 13)	Non-FAs group Controls (N = 138)	RR (95%CI) *P*-value	OR (95%CI) *P*-value
**Pre-conceptional blood sugar**			2.2	17.05
Controlled (N = 82)	1	81	(1.7-2.9)	(2.2-134.9)
Uncontrolled (N = 69)	12	57	<0.001*	0.007*
**HbA1c in first trimester**			2.8	24.8
<6.5 (N = 94)	1	93	(2.1 – 3.8)	(3.1-196.7)
≥6.5 (N = 57)	12	45	<0.001*	0.002*

*: Significant difference. GDM: Gestational diabetes mellitus. FAs: Fetal anomalies. HbA1c: Glycosylated hemoglobin. N: Number. OR: Odds ratio. RR: Relative risk.

## Discussion

157 pregnant women with GDM attending the antenatal clinic were included in this study, and data of 151 (96.2%) were finally analyzed to detect the prevalence of FAs in women with GDM (primary outcome), and the risk of FAs in relation to the pre-conceptional blood sugar and HbA1c (secondary outcome).

In this study, 63.6% (96) of the studied women had a positive family history of DM, and 30.5% (46) infants were delivered by caesarean section.

Allen et al. concluded that a careful history obtained from women with GDM can identify other risks, such as family history of DM or advanced maternal age, that may further increase the risk of chromosomal abnormalities or FAs [5].

Blackwell et al. concluded that the overall cesarean delivery rates for women with GDM Class A2, B, C, D-F pregnancies were 20.3%, 40%, 37%, and 57.1%, respectively [12].

FAs were recorded in 8.6% (13) of the studied women with GDM. The recorded FAs were cardiovascular [2.6% (4)], musculoskeletal [1.3% (2)], urogenital [1.3% (2)], gastrointestinal [1.3% (2)], facial [0.7% (1)], central nervous system [0.7% (1)], and multiple FAs [0.7% (1)].

Similarly, Aberg et al. found that the FAs associated with diabetic pregnancies included cardiovascular (TGA, VSD, situs inversus, and hypoplastic left ventricle); central nervous system (anencephaly, encephalocele, and spina bifida); caudal regression syndrome; renal agenesis; multicystic dysplasia; and gastrointestinal (anal/rectal atresia and small left colon) anomalies [13].

An association between DM and FAs has been recorded since the 19^th^ century. Caudal regression syndrome has had a strong association with DM (200 times more frequently in diabetic mothers than controls), and 4% of fetuses of diabetic women had at least one major FA (most commonly, CVS or musculoskeletal anomalies) [14].

Studies of infants of diabetic mothers showed increased risk of cardiovascular, genitourinary, musculoskeletal, and other FAs in women with uncontrolled diabetes during pregnancy [5, 6]. FAs are closely related to the HbA1c level in pregnancy [9, 10].

In this study, the uncontrolled pre-conceptional blood sugar significantly increased RR (RR 2.2; *P* < 0.001), and odds of FAs (OR 17.05; *P* = 0.007) in women with GDM. In addition, HbA1c ≥6.5 significantly increased RR (RR 2.8; *P* < 0.001), and odds of FAs (OR 24.8; *P* = 0.002) in women with GDM.

Miller et al. found the FAs rate was 22.4% in diabetic pregnant women when the maternal first trimester HbA1c was >8.5 [15]. Allen et al. concluded that pregnancy in diabetic women should be planned with proper pre-pregnancy counseling and proper glycemic control [5]. Previous studies and meta-analysis concluded that pre-conceptional counseling for diabetic women with optimum glycemic control can significantly decrease the risk of FAs [16, 17].

Baptiste-Roberts et al. demonstrated that women without diabetes before pregnancy are not at risk of FAs, and that an elevated HbA1c in the first trimester of pregnancy is a fair indicator of hyperglycemia during organogenesis [18]. In addition, the aneuploidy and chromosomal anomalies that occur with GDM are likely associated with increased maternal age [19, 20].

The Canadian Diabetes Association also concluded that type 1 or type 2 pregnant diabetic women should maintain the preconception HbA1c ≤7% to decrease the risk of FAs [8].

Miller et al. concluded that the HbA1c level before the 14^th^ week of gestation reflects the diabetic state during organogenesis and strongly correlated with the rate of FAs [15]. The rate of FAs was 3.4% when the maternal first trimester HbA1c was ≤8.5, and it was 22.4% when the maternal first trimester HbA1c was >8.5 [15].

Failure to study the chromosomal anomalies associated with the detected FAs, and absence of a control group (i.e. non-diabetic pregnant women), were the limitations of this study.

To the best of our knowledge, this study was the first prospective cohort study conducted in our region to detect the prevalence of FAs in GDM and the risk of FAs in relation to the pre-conceptional blood sugar and HbA1c. Future comparative studies, including the neonatal outcome of diabetic pregnant women compared to non-diabetic pregnant controls, are needed.

### Conclusion

In this study, the prevalence of FAs in women with GDM was 8.6%. Uncontrolled pre-conceptional blood sugar and HbA1c ≥6.5 in the first trimester significantly increased the relative risk and the odds of FAs.
